# Serum ADMA concentration – an independent factor determining FMD impairment in cardiac syndrome X

**DOI:** 10.3109/03009730903225537

**Published:** 2009-12-08

**Authors:** Maciej Haberka, Katarzyna Mizia-Stec, Zbigniew Gąsior, Magdalena Mizia, Joanna Janowska, Michał Holecki, Barbara Zahorska-Markiewicz

**Affiliations:** ^1^Department of Cardiology, Medical University of Silesia, KatowicePoland; ^2^Department of Pathophysiology, Medical University of Silesia, KatowicePoland

**Keywords:** Asymmetric dimethylarginine, cardiac syndrome X, flow-mediated dilatation, nitric oxide, platelet-derived growth factor

## Abstract

Mechanisms of decreased endogenous vascular reactivity in individuals with cardiac syndrome X (CSX) are not fully understood.

**Aim:**

To evaluate the following serum markers: total nitric oxide (NO), asymmetric dimethylarginine (ADMA), platelet-derived growth factor (PDGF), and to establish their relation to ultrasound indexes of endothelial function and structural remodeling in CSX patients.

**Method:**

The study group consisted of 43 CSX patients (mean age: 56.3 ± 9 years), while the control group included 21 healthy subjects (mean age: 54.86 ± 6.9 years). The high-resolution ultrasound was performed to measure: flow-mediated vasodilatation (FMD), nitroglycerine-mediated vasodilatation (NMD) and intima-media thickness (IMT) of carotid arteries.

**Results:**

In CSX patients, significantly lower FMD (9.06 ± 3.2%) and significantly higher IMT (0.667 ± 0.14 mm) values were observed compared to healthy individuals (17.42 ± 8.4%, 0.571 ± 0.2 mm; *P* < 0.05). Mean total NO serum concentration was significantly higher in the CSX group (48.2 ± 18.2 μmol/L) as compared to controls (32.1 ± 1.4 μmol/L; *P* < 0.0001). There were no differences in serum ADMA and PDGF levels. In CSX patients, FMD values correlated with NO (*r* = 0.323; *P* = 0.039) and ADMA (*r* = -0.387; *P* = 0.012) serum levels; however, there were no significant correlations between NO and ADMA concentrations.

**Conclusion:**

Serum ADMA concentration is the only independent factor determining FMD impairment.

## Introduction

Cardiac syndrome X (CSX) is usually regarded as a combination of typical, anginal chest pain and positive exercise tolerance test (ETT), but neither flow-limiting stenoses nor spasm on coronary angiography. A large body of evidence suggests its multifactorial pathophysiology with prevailing mechanisms, including subangiographic atherosclerosis, microvascular ischemia, and abnormal sensitivity to visceral pain (1). Literature data confirmed impaired endothelial function in individuals with CSX (2,3). According to some data the impairment of endothelial function assessed by flow-mediated dilatation (FMD) in CSX patients may be even comparable to that observed in patients with overt atherosclerosis, e.g. in coronary artery disease (CAD). The degree of FMD impairment may be important, as some authors suggest that CSX patients with more exaggerated endothelial dysfunction are characterized by a relatively worse prognosis (4).

The mechanisms of decreased endogenous vascular reactivity in individuals with CSX are not fully understood. Hence, we followed our interest in potential relationships among well documented biochemical markers of endothelial dysfunction or atherosclerosis and ultrasound vascular indexes: FMD and intima-media thickness (IMT).

The aim of our study was to evaluate the following serum markers: total nitric oxide (NO; measured as the stable end-product of NO, i.e. nitrite/nitrate), asymmetric dimethylarginine (ADMA; an endogenous competitive inhibitor of NO synthase), platelet-derived growth factor (PDGF) and to establish their relation to ultrasound indexes of endothelial function and structural remodeling in CSX patients.

## Material and methods

In total, 43 consecutive patients fulfilling the commonly recognized criteria for CSX (anginal chest pain, positive ETT, and angiographically normal coronary arteries) were included in the study (mean age: 55.9 ± 9.3 years; female/male: 29/14). The age- and sex-matched control group (mean age: 54.86 ± 6.9 years; female/male: 14/7) consisted of 21 healthy subjects without clinical evidence of angina confirmed in accessory investigations (ETT and/or coronary angiography).

The exclusion criteria were: acute and chronic inflammatory diseases (in 3 preceding months), cigarette smoking within 12 h before examination, diabetes mellitus, second and third degree of hypertension according to European Society of Cardiology guidelines, history of myocarditis and vasculitis, spondyloarthritis, Tietze's syndrome, gastrointestinal tract diseases, diseases of the aorta, hormone replacement therapy, arrhythmias that might disturb the multislice computer tomography evaluation (atrial fibrillation, increased number of ventricular extrasystolic beats, sinus tachycardia).

The clinical characteristics of patients consisted of: medical history (familial diseases, concomitant diseases, pharmacotherapy used, and smoking status), physical examination (arterial pressure, heart action, body mass, and height, waist, and hip circumference), laboratory tests (mainly lipidograms and fasting glucose), and accessory investigations (electrocardiogram, echocardiography, ultrasound vascular imaging) (Table I).

**Table I.** Participant characteristics.

	CSX (*n* = 43)	Controls (*n* = 21)	*P*
	Mean ± SD or *n* (%)	Mean ± SD or *n* (%)	
Age (years)	55.9 ± 9.3	54.86 ± 6.9	NS
Female / male	29 (67.4%) / 14 (32.6%)	14 (66.7%) / 7 (33.3%)	NS
Weight (kg)	82.1 ± 13.4	76.75 ± 14.7	NS
BMI (kg/m^2^)	30.1 ± 4.4	28.83 ± 4.7	NS
WHR	0.88 ± 0.1	0.87 ± 0.1	NS
Waist (cm)	96.9 ± 10.6	91.23 ± 12.9	NS
Heart rate (bpm)	64.07 ± 9.3	66.92 ± 12.2	NS
SBP (mmHg)	139.44 ± 22.7	124.66 ± 14.2	NS
DBP (mmHg)	82.22 ± 12.5	76.00 ± 9.5	NS
Hypertension	30 (69.8%)	0	<0.001
Smoking status:			
Current	3 (7.0%)	2 (9.5%)	NS
Ex-smoker	17 (39.5%)	6 (28.6%)	NS
Never smoked	23 (53.5%)	13 (61.9%)	NS
TCh (mg%)	203.3 ± 46.7	199.02 ± 39.2	NS
TG (mg%)	143.6 ± 89.6	136.44 ± 104.3	NS
HDL (mg%)	63.7 ± 16.9	60.02 ± 9.4	NS
LDL (mg%)	113.0 ± 39.0	117.33 ± 32.2	NS
LVEF (%)	60.5 ± 6.1	62.8 ± 7.2	NS
LVMI (g/m^2^)	128.0 ± 28.7	116.23 ± 26.7	NS

The diagnosis of hypertension was determined based on blood pressure (BP) levels (systolic BP ≥ 140 mmHg or diastolic BP ≥ 90 mmHg) or report of a prior diagnosis of hypertension and current antihypertensive treatment. In the study group, there were 30 CSX patients (69.8%) with mild hypertension (grade I), all of whom were treated with low or medium doses of angiotensin-converting enzyme inhibitors (ACE-I).

Echocardiography was performed in all patients according to the standards of the European Society of Echocardiography. The values of left ventricular ejection fraction (LVEF) and left ventricular mass index (LVMI) were subjected to analysis.

All clinical data were obtained at the time of the study. The measurements were performed after an overnight fast and 12–24 hours without hypertension medications and smoking.

The following vascular ultrasonography techniques were used to assess the functional and structural remodeling of the vascular system:

### FMD–flow-mediated dilatation

Endothelium-dependent flow-mediated dilatation (FMD) was assessed according to standard techniques (5). Measurements of brachial artery FMD were done in a quiet, temperature-controlled room, between 9 and 11 a.m. Patients were examined after at least a 10-minute rest; the ultrasound examination was performed in a supine position. Expert investigators took measurements in a B-mode presentation. The brachial artery of the dominant forearm was visualized above the antecubital fossa in a longitudinal plane, with a sphygmomanometric cuff on the proximal portion of the arm. The brachial artery diameter was described as a minimal distance between ‘m’ lines, from the anterior to the posterior wall of the artery. Images were acquired with an electrocardiography gating, with measurements made in end diastole, which corresponds to the onset of the R wave. The study was performed in three stages: Stage 1) base-line brachial artery diameter (BAd) and flow measurements were made, and an average was calculated for each subject; Stage 2) the sphygmomanometer cuff was inflated to 200 mmHg to occlude arterial inflow for 3 minutes; and Stage 3) brachial artery diameter and blood flow were measured and the mean calculated of the values obtained during 50–60 seconds after cuff deflation. Taking these two measurements into consideration (base-line and after cuff deflation), %FMD was calculated (percent increase of the artery diameter in comparison to base-line results). After a 10-minute rest, a sublingual tablet of nitroglycerine (0.5 mg) was administered to determine the maximum obtainable exogenous vasodilatory response. Brachial artery diameter and blood flow were measured following nitroglycerine (NTG), and %NMD was determined (NTG-induced percent increase of the artery diameter).

### IMT–intima media thickness

All measurements were performed in the common and internal carotid arteries. The common and internal carotids were studied in longitudinal planes with anterior and lateral approaches. IMT was measured within the posterior wall of the artery. The average of ten measurements was used to calculate IMT.

Expert investigators performed the echocardiography and the vascular ultrasound measurement using a high-frequency ultrasound system (Toshiba Aplio) equipped with a 3.5 MHz sector probe, a high-frequency vascular transducer (multiple frequency: 7–10 MHz), and software for two-dimensional (2D) imaging, color and spectral Doppler, an internal electrocardiogram monitor.

### Blood sampling and laboratory measurements

Blood samples were collected from each subject after a 10-hour fast. After centrifugation, aliquots were frozen at -80° C until assayed.

The measurements of total NO/nitrite/nitrate were performed with the total NO/nitrite/nitrate assay kit (R&D Systems, Inc., Minneapolis, USA), based on the enzymatic conversion of nitrate to nitrite by reductase, followed by colorimetric detection of nitrite (Griess reaction). Human PDGF-BB measurements were done with the use of enzyme-linked immunosorbent assay (ELISA) kits (R&D Systems, Inc.). The sensitivity of the total NO/nitrite/nitrate assay was 0.25 μmol/L. Intra- and interassay coefficients of variation for total NO/nitrite/nitrate were <2.5% and <4.6%, respectively. The sensitivity limit for PDGF-BB was 15 pg/mL. The intra-assay coefficient of variation was <4.5%, and the interassay coefficient of variation was <7.6%.

The serum ADMA concentration was assessed by the ADMA ELISA kit (Immundiagnostik AG, Bensheim, Germany). The ADMA test disclosed values as low as 0.05 μmol/L. The intra-assay coefficient of variation was <9.8%, and the interassay coefficient of variation was <7.5%.

Lipid parameters (serum cholesterol, HDL-cholesterol, LDL-cholesterol, triglycerides) were measured using commercially available test kits (Point Scientific Inc., Michigan, USA).

### Statistical analysis

All text and table results are expressed as means ± SD or number and percentage. The results were analyzed with the ANOVA test, including the Newman-Keuls correction. Clinical parameters and the results of accessory investigations were compared using the chi-square test for proportions with the Yates correction, the two-sample *t* tests for normally distributed continuous variable (Student's *t* test); in case of abnormal distribution, the Mann-Whitney U test was used. Multivariable logistic and linear regressions were used to assess independent predictors of the FMD and IMT, respectively. A value *P* < 0.05 was considered statistically significant.

## Results

### Clinical characteristics

The clinical characteristics are set out in Table I.

### Vascular parameters

Vascular indexes evaluation in cardiac syndrome X patients revealed significantly lower FMD (9.06 ± 3.2%) and significantly higher IMT (0.667 ± 0.14 mm) values compared to healthy individuals (17.42 ± 8.4%, 0.571 ± 0.2 mm; *P* < 0.05), whereas brachial artery diameter (BAd) and nitroglycerine-mediated vasodilatation (NTG-MD) were comparable between both groups (Table II).

**Table II.** Results.

	CSX (*n* = 43)	Controls (*n* = 21)	*P*
	Mean ± SD	Mean ± SD	
BAd (mm)	4.00 ± 0.6	4.01 ± 0.5	NS
FMD (%)	9.06 ± 3.2	17.42 ± 8.4	0.008
NMD (%)	23.26 ± 13.4	24.95 ± 13.4	NS
IMT (mm)	0.67 ± 0.2	0.57 ± 0.2	0.021
ADMA (μmol/L)	0.41 ± 0.08	0.41 ± 0.13	0.98
PDGF (pg/mL)	5193.6 ± 2530.1	4716.9 ± 932.4	0.368
Total NO (μmol/L)	48.2 ± 18.2	32.1 ± 1.4	<0.0001
	range: 30.1--107.9	range: 30.3--34.2	

### Biochemical markers

Among biochemical serum markers, only total NO serum concentrations were significantly higher in the CSX group compared to the control group (48.2 ±18.2 versus 32.1 ± 1.4 μmol/L; *P* < 0.0001), while no significant differences between both groups were found in relation to ADMA (0.41 ± 0.08 versus 0.41 ± 0.13 μmol/L; *P* = 0.98) and PDGF (5193.62 ± 2530.1 versus 4716.95 ± 932.36 pg/mL; *P* = 0.368) serum levels (Table II).

### Correlation analysis

In the CSX group, total NO serum concentrations correlated significantly with FMD (*r* = 0.323; *P* = 0.039) and inversely with IMT (-0.354; *P* = 0.023) values. Moreover, ADMA serum levels correlated significantly with IMT (0.384; *P* = 0.023) and inversely with FMD (0.387; *P* = 0.012) values. Multivariate regression analysis revealed the ADMA concentration (*t* = -2.925; *P* = 0.007) and base-line brachial artery flow velocity (*t* = 3.965; *P* = 0.001) as independent factors determining the FMD value (*P* = 0.001), whereas PDGF serum concentrations were found to correlate only with IMT values (*r* = 0.333; *P* = 0.038).

There were no associations between total NO, ADMA, and PDGF concentrations.

## Discussion

The body of evidence supports the hypothesis of early functional and structural vascular changes in the course of CSX. In our study, impaired vascular indexes, including lower FMD and higher IMT, were shown in the CSX group. Impaired FMD and increased IMT constitute well evidenced markers of early atherosclerosis and are common findings in most CSX individuals observed in the previous studies (6–8). It is of great importance that non-invasive ultrasound brachial artery FMD assessment is in close relation to coronary artery endothelium-dependent vasomotor responses to acetylcholine (9). Most studies showed that oral L-arginine administration is followed by FMD improvement in CSX patients, suggesting generalized endothelial dysfunction related to nitric oxide (NO) activity diminution (10–12). However, there are studies providing conflicting observations and showing preserved microcirculatory endothelial function and impaired adenosine-mediated endothelium-independent vasodilatation in CSX patients (10).

In the present study, we assessed whether biochemical markers may provide any new information to the vascular indexes and the mechanism of vascular abnormalities in CSX patients.

There are conflicting results in the literature on NO and ADMA in CSX patients. Most studies confirm elevated ADMA and decreased NOx plasma levels in the CSX population (1–18). ADMA plasma levels were associated with impaired FMD (19), and recently an association with impaired myocardial tissue perfusion was demonstrated (20). The results of our study were not in accordance with the above-mentioned data. We showed similar ADMA and even increased NOx plasma levels in CSX patients compared to normal controls. Plasma NOx levels revealed a positive correlation with FMD and an inverse association with IMT.

Regardless of the higher NO levels in CSX, the FMD values were decreased as compared to controls. It should be noted that NO analysis concerned the base-line NO levels only. Thus we might conclude only that base-line NO levels were higher in CSX as compared to controls. At the same time, the FMD marker of both shear-stress-induced NO release and its bioavailability was impaired. The positive correlation between basal NO level and FMD suggested that in patients with higher base-line NO level the NO release was higher. Vascular responsiveness to NO was impaired and higher NO concentrations might have contributed to the inappropriate adaptive mechanism. Our confusing findings on NO levels might also be related to the significant presence of arterial hypertension in the CSX group (30 patients; 69.8%) and ACE-I treatment. Besides, increased concentrations of NO levels might reflect an increased generation of oxidants and augmented oxidative stress.

In the literature, there are few studies that do not confirm decreased NO levels in CSX subjects. Soysal et al. showed substantially decreased NOx plasma levels (20.2 μmol/L) in CAD patients, while similar plasma levels (33.4 μmol/L versus 35 μmol/L) were observed in CSX patients and normal controls (21). In another study, Fragasso et al. found mildly decreased NOx levels in CSX patients compared to normal controls (24.2 μmol/L versus 26.8 μmol/L) (22). Moreover, Desideri et al. did not demonstrate impaired endothelial function in CSX patients in terms of base-line plasma concentrations of NOx, endothelin-1, and soluble vascular cell adhesion molecule 1 (VCAM-1) (23).

PDGF is another marker assessed in our study. It plays a crucial role in the proliferation of different cell types. Given that increased expressions of PDGF and its receptors within atherosclerotic lesions were observed, PDGF has been implicated in an inflammatory-fibroproliferative process. Furthermore, PDGF has been demonstrated to have an angiogenic effect, affect blood vessel tonus by inducing vascular cell constriction, and on the other hand NO-mediated relaxation and finally platelet aggregation (24). There are lots of clinical trials on the role of PDGF in CAD and its association with cardiovascular risk factors (25–32). The data on PDGF in CSX are limited. In our study, we found comparable PDGF plasma concentrations between CSX patients and normal controls. However, a significant positive correlation with IMT was demonstrated, which confirms PDGF association with vascular wall thickening.

The biochemical markers analyzed correlated with ultrasound vascular indexes. We found the associations in terms of NOx and ADMA to both indexes, and as mentioned above between PDGF and IMT. However, among CSX patients, plasma ADMA concentrations were found to be an independent factor determining only FMD values. It suggests the great importance of ADMA in the pathophysiology of CSX.

Individuals with CSX have been assigned excellent long-term prognoses. However, recent studies suggest that the CSX population is not homogenous, and a subpopulation with endothelial dysfunction might be attributed a worse prognosis than previously believed. Further observations of the CSX group might explain the importance of lower FMD values and the association with a new risk marker–ADMA serum level.

There are some limitations of the study. The FMD values are influenced by different factors as previously described (33). In order to avoid disturbances in FMD evaluation we established the inclusion and exclusion criteria and performed examinations in optimal conditions. There are different methods of NO serum level/bioavailability analysis. Therefore, inconsistencies in study results might be partially explained by different methodologies. Various cellular sources of PDGF measured in human plasma must be considered before final conclusions are drawn. Moreover, PDGF concentrations measured in peripheral circulation might differ from local plasma levels in the coronary circulation (34). The role of PDGF and its relation to atherosclerotic clinical complications still need to be clarified. Finally, systemic arterial hypertension, with a relatively high occurrence in the study group of antihypertensive treatment with ACE-I, might have affected the obtained levels of biochemical parameters, including NOx plasma concentrations. Although only patients with arterial hypertension grade I were enrolled, as according to the new CSX definition (1), the concomitance of left ventricle hypertrophy and advanced hypertension excludes a CSX diagnosis. The lack of LVMI differences between the CSX group and controls confirms our statement.

In summary, CSX patients were characterized by early functional and structural markers of vascular remodeling. Biochemical analysis revealed increased serum NOx levels in this group as compared to healthy subjects. Both the FMD index of endothelial function and IMT correlated with biochemical markers of NO bioavailability. However, serum ADMA concentration was the only independent factor determining the FMD impairment. The above observations constitute a next step in better recognition of CSX pathophysiology. Further studies are needed to verify their clinical significance.
